# Pulsed laser synthesis of free-standing Pt single atoms in an ice block for enhancing photocatalytic hydrogen evolution of g-C_3_N_4_†

**DOI:** 10.1039/d5na00043b

**Published:** 2025-03-26

**Authors:** Yongming Fu, Qianyu Lu, Jianhong Wang, Na Sun, Jinjun Gao, Peng Chen, Jizhou Wu, Jie Ma

**Affiliations:** a School of Physics and Electronic Engineering & Institute of Laser Spectroscopy, State Key Laboratory of Quantum Optics and Quantum Optics Devices, Shanxi University Taiyuan 030006 China fuyongming@sxu.edu.cn mj@sxu.edu.cn; b Xinzhou Institute of Innovation Ecosystem, Shanxi University Xinzhou 034000 China; c Collaborative Innovation Center of Extreme Optics, Shanxi University Taiyuan 030006 China

## Abstract

This study reports an innovative synthesis method of a Pt/g-C_3_N_4_ single atom catalyst for enhancing photocatalytic hydrogen evolution. The method involves the synthesis of free-standing Pt single atoms within an H_2_PtCl_6_ ice block using a pulsed laser reduction process, followed by transferring them onto few-layer g-C_3_N_4_ through electrostatic adsorption at low temperature. This approach eliminates the need for high-energy lasers and porous support materials during laser solid-phase synthesis. The photocatalytic activities of Pt/g-C_3_N_4_ synthesized under various laser conditions are evaluated to optimize the synthesis parameters. The optimal Pt/g-C_3_N_4_ catalyst demonstrates a significantly higher photocatalytic hydrogen evolution capability (320 μmol h^−1^), 129 times that of pure g-C_3_N_4_ (2.2 μmol h^−1^). This work expands the laser-solid phase synthesis method, offering a promising route for the production of single atom catalysts with simple operation, clear synthetic pathways, low cost, and environmental friendliness.

## Abstract 2

This study developed a novel method for synthesizing single atom catalysts to boost photocatalytic hydrogen production, involving pulsed laser reduction of Pt single atoms in an ice block and low-temperature adsorption on few-layer g-C_3_N_4_.

## Graphical abstract

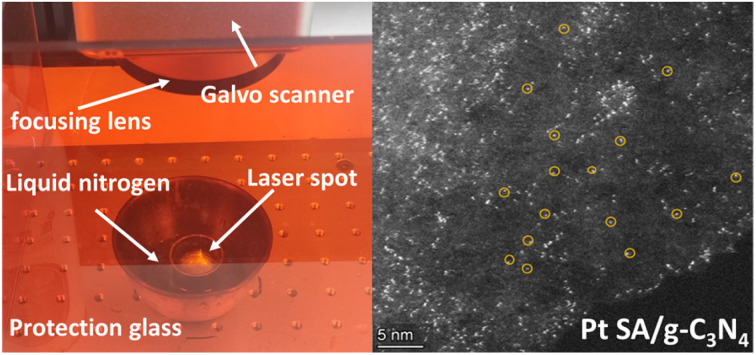

## 1. Introduction

Single atom catalysts (SAC) have garnered significant attention for various photocatalytic and electrocatalytic applications, including CO_2_ reduction, water splitting, CO oxidation, and N_2_ fixation.^1–3^ In SAC, isolated atoms are anchored on the surface of a support material through electron exchange interactions, forming dispersed and localized active sites. The distinctive structure of SAC allows for precise control over catalytic reactions at the atomic level, maximizing catalytic efficiency, selectivity, and atomic utilization.^4^ Despite their notable advantages, the most significant challenge in SAC is the aggregation of single atoms arising from their high surface free energy, which significantly compromises their unique catalytic properties and ultimately limits the practical applications of SAC.^5^

To address this challenge, researchers are actively exploring innovative approaches to enhance the dispersion of single atoms. One effective approach is the spatial confinement strategy, which involves the encapsulation of single atoms within molecular cages to prevent their migration.^6^ Unlike the traditional spatial confinement techniques that depend on the use of porous materials, Wu *et al.* proposed a novel ice-phase confinement method.^7^ In this approach, Pt ions in an H_2_PtCl_6_ aqueous solution are stabilized and dispersed by forming H–Pt–OH complexes with water molecules. The solution is subsequently frozen into ice blocks, where the crystalline structure of water molecules restricts the thermal movement of Pt atoms during the photoreduction process. Although this innovative technique effectively suppresses the aggregation of Pt single atoms, the low photon density of conventional light sources, such as mercury lamps, limits the quantity of synthesized Pt single atoms.

Laser reduction methods have been developed for one-step synthesis of SAC, capitalizing on the localized high temperature and rapid cooling effects generated by high-energy laser pulses interacting with the support material.^8–12^ The high photon density in a single laser pulse significantly increases the probability of interactions between photons and metal precursors, thereby enhancing the yield of single atoms. Furthermore, the brief duration of the laser pulse limits the formation of single atoms at any given time, effectively minimizing the aggregation and nucleation of single atoms. For instance, Liu *et al.* proposed a laser solid-phase synthesis technique that facilitates the simultaneous reduction of metal precursors and graphene oxide in one step.^10^ Zou *et al.* introduced a laser planting strategy in which high-energy laser pulses decompose metal precursors into single atoms while concurrently inducing vacancy defects on the surface of support materials for anchoring single atoms through electrostatic interactions.^13^ Compared to the conventional photochemical reduction methods, laser reduction methods eliminate the need for high-temperature treatments and additional reductants, enabling the one-step synthesis of high-loading metal single atoms under mild conditions. However, the laser synthesis methods rely on high pulsed energy to introduce vacancy defects on the support material surface for stabilizing single atoms, resulting in a strong dependence on the high-power laser and the specific characteristics of the support material.

In this letter, we present an innovative method for synthesizing SAC by integrating the freeze-phase photochemical method with the laser reduction technique. Free-standing Pt single atoms are synthesized within the H_2_PtCl_6_ ice block through a pulsed laser reduction process, followed by transferring them onto few-layer g-C_3_N_4_ at low temperature. The 355 nm pulsed laser directly induces the reduction of the Pt precursor, eliminating the need for high-energy lasers and porous support materials. Freezing the precursor solution facilitates the confinement of metal atoms by water molecules, ensuring stable dispersion and preventing nucleation during the formation of Pt single atoms. Two-dimensional g-C_3_N_4_ with an intrinsic six-fold cavity is chosen as the support material,^14^ enabling the electrostatic adsorption of Pt single atoms by Pt–N interaction and further suppressing atom agglomeration. Furthermore, the gradual thawing of the ice block reduces the concentration of dissociative single atoms in the suspension, promoting their successful anchoring onto the g-C_3_N_4_. The photocatalytic hydrogen evolution capabilities of Pt single atoms anchored on g-C_3_N_4_ (Pt/g-C_3_N_4_) synthesized under different conditions are evaluated to investigate the effect of the pulse repetition frequency and scanning speed. This method offers a promising route for the production of SAC, featuring simple operation, clear synthetic pathways, low cost, and environmental sustainability.

## 2. Experimental

Free-standing Pt single atoms were directly synthesized through a laser freeze-phase reduction method using a home-made laser scanning set (Fig. S1†). 10 mL of H_2_PtCl_6_ aqueous solution (1 mg mL^−1^) was transferred to a stainless-steel cup with a diameter of 3 cm and rapidly frozen in liquid nitrogen to form a cylindrical ice block with a thickness of 20 mm (Fig. S2a†). The ice block was then placed under a commercially available low-power pulsed laser (P-Plus2-355-5, Huaray, China) with a wavelength of 355 nm. The laser was set at a current of 1 A, a repetition frequency of 50 kHz, and a pulse width of 50 ns. A lens with a focal length of 190 mm was used to focus the laser beam onto the upper surface of the ice block, achieving a focused spot diameter of 25 μm (Fig. S2b†). A two-dimensional galvo scanner was employed to control the laser spot for continuous horizontal scanning throughout the ice block surface at a scanning speed of 700 mm s^−1^. To ensure the entire volume of the ice block was exposed to the laser rather than just at the surface, a linear stage was used to control the vertical scanning of the laser beam with a speed of 28 mm min^−1^. To compensate the power loss at greater depths caused by the absorption of the ice block (Fig. S3†), multiple repeated scans were conducted on the ice block from top to bottom. During the scanning process, the ice block was maintained in liquid nitrogen to prevent melting. After a scanning duration of 140 min, free-standing Pt single atoms were obtained in the ice block.

The Pt single atoms were anchored onto few-layer g-C_3_N_4_ through strong electrostatic adsorption at low temperature. Bulk g-C_3_N_4_ powder was synthesized by thermal polymerization of urea. A few-layer g-C_3_N_4_ suspension was prepared by dispersing 50 mg of bulk g-C_3_N_4_ powder in 10 mL of deionized water, followed by ultrasonic treatment at a power of 400 W for 2 h. The ice block containing Pt single atoms was put into the suspension under vigorous stirring for 48 h. The temperature was maintained at 2 °C to slow the ice melting. Subsequently, the 20 mL suspension was centrifuged 5 times to remove any residual Pt species. Finally, the resulting Pt/g-C_3_N_4_ powder was dried using a freeze-drying process.

The Pt content in Pt/g-C_3_N_4_ was quantified using inductively coupled plasma-optical emission spectroscopy (ICP-OES, 5510, Agilent, USA). The surface morphology was characterized with a transmission electron microscope (TEM, JEM-F200, JEOL, Japan). The identification of crystalline phases, chemical functional groups, and surface chemical states was performed using X-ray diffraction (XRD, Rigaku Miniflex 600, Japan), Fourier transform infrared spectroscopy (FTIR, Nicolet iS20, Thermo Scientific, USA), and X-ray photoelectron spectrometry (XPS, K-Alpha, Thermo Scientific, USA), respectively. Diffuse reflectance spectroscopy (DRS, UH5700, Hitachi, Japan) was employed to acquire the UV-visible absorption spectroscopy data. The steady and time-resolved photoluminescence (PL) spectra were measured using a photoluminescence spectrometer (FLS1000, Edinburgh, UK). High angle annular dark-field scanning transmission electron microscopy (HAADF-STEM, JEM-ARM200F, JEOL, Japan) images were captured to confirm the atomic dispersion of Pt atoms. X-ray absorption fine spectroscopy (XAFS) measurement of the Pt element was conducted at the XAFS beamline of the Shanghai Synchrotron Radiation Facility (SSRF), where the experimental data were acquired in fluorescence mode using an ionization chamber.

The photocatalytic water splitting reactions were conducted using a photocatalytic activity evaluation system (CEL-PAEM-D8Plus, CEAULIGHT, China). In a typical experiment, 50 mg of Pt/g-C_3_N_4_ catalyst was suspended in 80 mL of deionized water and 20 mL of triethanolamine. Prior to the photocatalytic test, the closed-loop system was evacuated for 20 min to eliminate the air in the reaction system. The generated H_2_ gas was analyzed every 30 min using gas chromatography (CG-7920, CEAULIGHT, China) with Ar gas as the carrier gas. During the photocatalytic test, a 300 W xenon lamp was used to provide the simulated light source. The system was irradiated for 4 h to assess the H_2_ evolution performance of SAC. The reaction temperature was maintained at 4 °C using cooling water.

## 3. Results and discussion

**Fig. 1:**
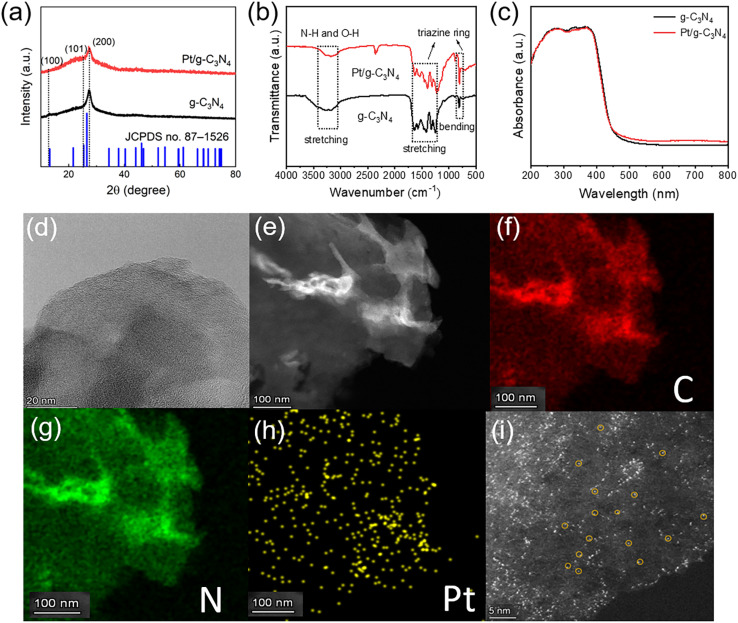
Microstructures of few-layer g-C_3_N_4_ and Pt/g-C_3_N_4_. (a) XRD patterns. (b) FTIR spectra. (c) DRS spectra. (d) High-resolution TEM image of Pt/g-C_3_N_4_. (e–h) Elemental mapping profiles of Pt/g-C_3_N_4_ under the dark-field of HAADF-STEM (e), C (f), N (g), and Pt (h). (i) High-resolution HAADF-STEM image under dark-field to clearly demonstrate Pt single atoms.

Fig. 1a presents the XRD patterns of few-layer g-C_3_N_4_ and Pt/g-C_3_N_4_. The characteristic diffraction peaks at 12.95°, 25.31°, and 27.38° correspond to the (100), (101), and (002) crystallographic planes of g-C_3_N_4_ (JCPDS file no. 87-1526), respectively.^15^ The diminished intensity and enhanced angle of the (002) peak suggests a reduced long-range order and enlarged interface distance in the stacking of g-C_3_N_4_ layers, which aligns with the characteristics of few-layer structure.^16^ The diminished intensity of the (002) peak suggests a reduced long-range order in the stacking of g-C_3_N_4_ layers, which aligns with the characteristics of few-layer structure. As depicted in the FTIR spectra (Fig. 1b), the two samples exhibit similar vibrational peak bands corresponding to N–H stretching (3100–3300 cm^−1^), C=N or C–N stretching (1200–1700 cm^−1^), and triazine breathing modes (811 cm^−1^) in heptazine rings, indicating that the introduction of Pt species does not significantly affect the molecular backbone of g-C_3_N_4_.^17^ DRS results demonstrate that the absorption intensity in the UV region of the Pt/g-C_3_N_4_ is comparable to that of few-layer g-C_3_N_4_ (Fig. 1c). By extrapolating the intercept of the *x*-axis in the linear region of Tauc plots, the band gaps of g-C_3_N_4_ and Pt/g-C_3_N_4_ are calculated to be 2.65 and 2.66 eV, respectively, suggesting that the bandgap is not effectively affected by Pt incorporation. The formation of Pt single atoms anchored on few-layer g-C_3_N_4_ is then morphologically studied. Fig. 1d is the TEM image of Pt/g-C_3_N_4_. There are no obvious Pt nanoparticles on the surface of few-layer g-C_3_N_4_. However, the homogeneous distribution of C, N, and Pt elements (Fig. 1e–h) suggests that trace amounts of Pt atoms exist in the form of isolated single atoms on the surface of g-C_3_N_4_, which is further demonstrated by the densely dispersed bright spots (Fig. 1i) in HAADF-STEM analysis under dark-field.^18^ These results verify the successful synthesis of free-standing Pt single atoms through the laser freeze-phase reduction method and subsequent transfer onto few-layer g-C_3_N_4_ through low-temperature electrostatic adsorption. The mass fraction of Pt single atoms in Pt/g-C_3_N_4_ is measured to be 2.37% by HAADF-STEM and 1.19% by ICP-OES, indicating a relatively high loading mass of Pt single atoms on few-layer g-C_3_N_4_.

**Fig. 2:**
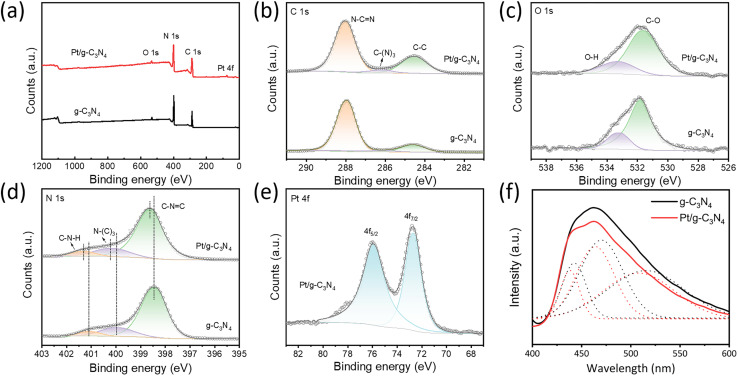
Chemical state of few-layer g-C_3_N_4_ and Pt/g-C_3_N_4_. (a–e) XPS spectra of survey (a), C 1s (b), O 1s (c), N 1s (d), and Pt 4f (e). (f) PL spectra.

The impact of Pt single atoms on the surface states of few-layer g-C_3_N_4_ is studied by XPS, as illustrated in Fig. 2. The survey spectra indicate the presence of C, N, and O in the g-C_3_N_4_ and Pt in the Pt/g-C_3_N_4_ (Fig. 2a). The high-resolution C 1s spectrum of g-C_3_N_4_ is well fitted to three distinct peaks at 284.6, 286.1, and 287.9 eV, corresponding to the C–C, C–(N)_3_, and N–C=N bonds, respectively (Fig. 2b). The g-C_3_N_4_ and Pt/g-C_3_N_4_ also show similar O 1s spectra, which are divided into two peaks at 531.82 and 533.18 eV, corresponding to the C–O and O–H bonds formed during the thermal polymerization process (Fig. 2c).^19^ The N 1s spectrum of g-C_3_N_4_ exhibits three peaks classified as pyridinic N (398.40 eV), pyrrolic N (399.95 eV) and amino groups (401.40 eV). For Pt/g-C_3_N_4_, the binding energies shift towards higher values (Fig. 2d), which is indicative of the formation of N coordination bonds with Pt single atoms.^20^ In the Pt 4f spectrum (Fig. 2e), the two symmetrical peaks located at 72.76 and 75.94 eV correspond to the 4f_7/2_ and 4f_5/2_ states of Pt species, suggesting an oxide state ranging between +2 and +4.^21^ PL spectra reveal that both few-layer g-C_3_N_4_ and Pt/g-C_3_N_4_ exhibit broad emission peaks within 400–600 nm (Fig. 2f). Deconvoluted by Gaussian fitting, the two spectra can be similarly divided into three emission centers belonging to pathways of π* → π, σ* → LP, and π* → LP transitions, respectively.^22,23^ A red-shift is observed in Pt/g-C_3_N_4_, indicating the bandgap narrowing of the sp^2^ C–N hybridize state. Moreover, the PL intensity of Pt/g-C_3_N_4_ is lower than that of g-C_3_N_4_, suggesting that the incorporation of Pt single atoms effectively suppresses the recombination of photogenerated charge carriers, thereby enhancing the photocatalytic performance.^24^

**Fig. 3:**
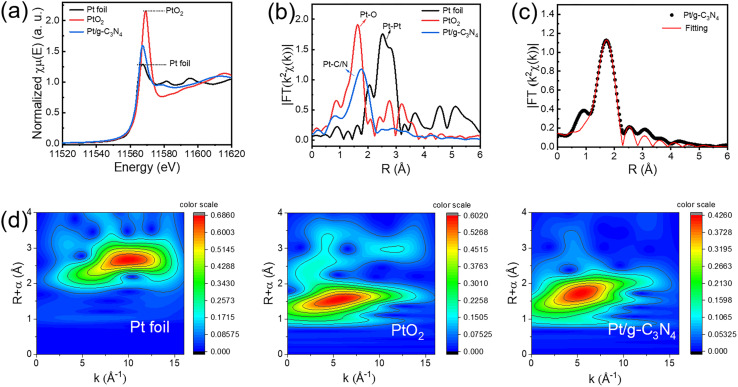
XAFS results of Pt/g-C_3_N_4_ compared with Pt foil and PtO_2_. (a) XANES spectra. (b) FT-EXAFS spectra. (c) FT-EXAFS fitting through Pt–N. (d) WT-EXAFS spectra.

The precise coordination environment and chemical state of Pt atoms in Pt/g-C_3_N_4_ are elucidated through synchrotron-based X-ray absorption near-edge structure (XANES) and extended X-ray absorption fine structure (EXAFS) (Fig. 3).^25^ The energy of the white line peak for Pt species in Pt/g-C_3_N_4_ is 11 567.5 eV, positioned between reference points of Pt foil and PtO_2_ (Fig. 3a and S4†). The Pt single atom interacts with the pyridinic N and facilitates the transfer of electrons from the Pt atom to g-C_3_N_4_, resulting in an oxidation state of Pt^*δ*+^ (0< *δ* < 4).^26^ The *k*^2^-weighted *R*-space Fourier transformed EXAFS (FT-EXAFS) spectrum of Pt/g-C_3_N_4_ exhibits a prominent peak around 1.77 Å, corresponding to the first-shell Pt–C/N scattering path (Fig. 3b and Table S1†). The absence of signals at 2.54 Å for the Pt–Pt bond and 1.62 Å for the Pt–O bond further confirms the Pt single atom feature, consistent with the previous HAADF-STEM observations. To ascertain the coordinating atom with the Pt single atom, the EXAFS spectrum of Pt/g-C_3_N_4_ is fitted, showing that the Pt single atom is anchored to g-C_3_N_4_ through the Pt–N pathway with a coordination number of 3.8 ± 0.2 and a bond length of 2.25 Å (Fig. 3c). The wavelet transformed EXAFS (WT-EXAFS) provides a method for simultaneously analyzing information in both *R*-space and *k*-space resolution.^27^ As shown in Fig. 3d, the maximum intensity of Pt/g-C_3_N_4_ centered round 5.42 Å^−1^ in *k*-space corresponds to a resolution of 1.77 Å in *R*-space, which is assigned to the Pt–N coordination structure. Note that the intensities centered at 10.08 and 5.06 Å^−1^ associated with Pt–Pt and Pt–O coordination are not observed in the Pt/g-C_3_N_4_. The coordination analyses substantiate the oxidation state of the Pt single atoms and their strong interaction with N atoms in an isolated state. The results indicate that the Pt single atoms synthesized by laser freeze-phase reduction are stably anchored in the six-fold cavities of g-C_3_N_4_ through electrostatic adsorption. The electron-rich N atoms in the g-C_3_N_4_ framework can provide sufficient coordination sites,^28^ and the unique structure with six-fold cavity provides most favorable anchored sites for embracing metal single atoms through the formation of metal–nitrogen bonds.^29–31^ Furthermore, the deformed wrinkle space of g-C_3_N_4_ helps in stabilizing single-atom Pt in the six-fold cavity.^32^ This anchoring mechanism is vital for maintaining the dispersion and stability of Pt single atoms, which is essential for enhancing the photocatalytic performance of Pt/g-C_3_N_4_.

**Fig. 4:**
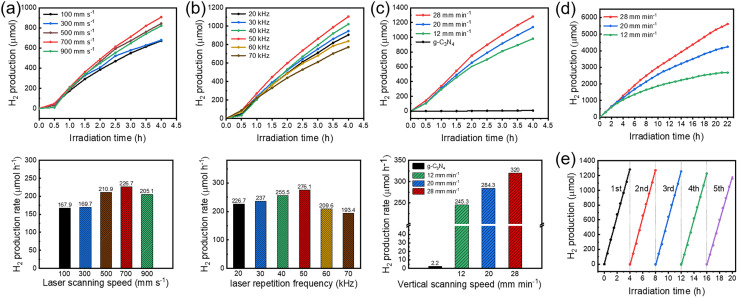
The photocatalytic hydrogen evolution performance of Pt/g-C_3_N_4_ under different synthetic conditions. (a) Effect of laser scanning speed. (b) Effect of laser repetition frequency. (c) Effect of vertical scanning speed. (d) Prolonged photocatalytic test over 22 h. (e) Photocatalytic capabilities within five cycles.

To comprehensively examine the influence of synthetic conditions on the photocatalytic hydrogen evolution performance of few-layer g-C_3_N_4_, the free-standing Pt single atoms are synthesized under different laser parameters. These parameters can be generalized by considering the energy density and pulse duration, which are critical factors in laser–material interactions. Based on previous studies involving pulsed laser synthesis of single atoms,^8,10,13^ three crucial parameters of horizontal scanning speed, vertical scanning speed, and pulse repetition frequency are focused on. The located energy density can be calculated based on the laser power, spot size, and scanning speed, while the pulse duration determines the thermal effects and cooling rates, which are critical for the synthesis of Pt single atoms. Under a pulse repetition frequency of 20 kHz, it is observed that the speed of 700 mm s^−1^ achieves the highest hydrogen production with horizontal scanning speeds ranging from 100 to 900 mm s^−1^ (Fig. 4a). The pulsed laser breaks the continuous horizontal scanning into a “point-to-point” scanning mode, which prevents a single scan from covering all Pt species within the ice block. The distance between adjacent points is mainly determined by the horizontal scanning speed. Given that the total scanning time is fixed to 140 min, a faster horizontal scanning speed results in more repeated scans, leading to a higher coverage rate of the laser spot on the Pt species, and consequently, an increased generation of Pt single atoms. However, excessively high speed may reduce the interaction time between the laser beam and the Pt precursor, negatively impacting the formation of Pt single atoms. Similarly, the pulse repetition frequency, evaluated from 20 to 70 kHz, reveals an optimal frequency of 50 kHz for maximizing hydrogen evolution (Fig. 4b). When the average power and pulse width of the pulsed laser remain constant, the pulse frequency is inversely proportional to the pulse energy. The formation of Pt single atoms primarily occurs during the pulse duration. A lower pulse frequency allows for a longer cooling time, resulting in reduced thermal accumulation of the laser, which helps to prevent the aggregation of Pt single atoms. However, excessively low frequencies result in excessively high pulse energy, intensifying the thermal motion of Pt atoms during a single pulse, which can lead to cluster formation. The vertical scanning speed is also investigated, indicating that a vertical scanning speed of 28 mm min^−1^ achieves the best photocatalytic performance (Fig. 4c). The vertical scanning speed is significantly lower than the horizontal speed, primarily influencing the spacing between Pt single atom layers. Due to the recoating effect of pulsed laser scanning, if the vertical scanning speed is too slow, the same ice layer may be scanned repeatedly, causing atom aggregation. Under the optimal conditions—700 mm s^−1^ for horizontal scanning speed, 28 mm min^−1^ for vertical scanning speed, and 50 kHz for pulse repetition frequency—the hydrogen production rate of the Pt/g-C_3_N_4_ reaches 320 μmol h^−1^, representing a remarkable increase of 129 times compared to that of pure g-C_3_N_4_ (2.2 μmol h^−1^), demonstrating a substantial enhancement in photocatalytic activity. The significant enhancement in photocatalytic hydrogen evolution is primarily attributed to the synergistic effects of improved electron transfer and increased reaction sites. Pt single atoms form Pt–N coordination with N atoms in g-C_3_N_4_, leading to the formation of N 2p–Pt 5d hybrid orbitals at the interface.^17^ The Pt–N coordination reduces the interfacial charge transfer resistance, which significantly suppresses the recombination of photogenerated electron–hole pairs.^33,34^ Moreover, the incorporation of Pt single atoms effectively lowers the Gibbs free energy for H* adsorption. When H* is adsorbed on the nitrogen atoms, the charge redistribution within the Pt–N coordination structure further decreases the Gibbs free energy for H* adsorption,^31^ providing more active sites for the photocatalytic hydrogen evolution reaction.

**Fig. 5:**
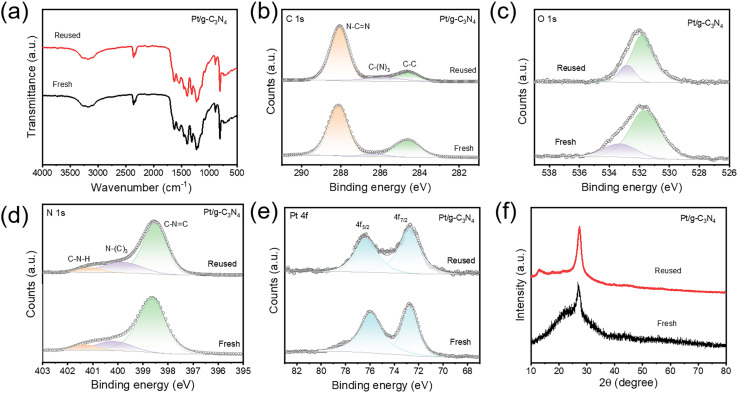
The characterization of Pt/g-C_3_N_4_ after five photocatalytic cycles. (a) FTIR spectra. (b–e) XPS spectra of C 1s (b), O 1s (c), N 1s (d), and Pt 4f (e). (f) XRD patterns.

It is worth noting that the concentration of Pt single atoms also affects the final loading mass. As the size of the ice block keeps constant and is strictly determined by the stainless-steel cup, the concentration of Pt single atoms is simply modulated by changing the H_2_PtCl_6_ concentration in the precursor solution. The photocatalytic hydrogen evolution of Pt/g-C_3_N_4_ with different Pt loading is shown in Fig. S5.† When the H_2_PtCl_6_ concentration increases from 0.1 to 1 mg mL^−1^, the rising photocatalytic activity is mainly due to the increased number of Pt single atoms by laser synthesis. Once the concentration exceeds 1 mg mL^−1^, the decreasing photocatalytic activity may be attributed to two possible reasons. One is that the excessive H_2_PtCl_6_ concentration enhances the absorption of the UV laser, resulting in lower generation of Pt single atoms in the ice block. The other possibility is that an excessive number of Pt single-atoms synthesized within the ice tend to form clusters due to extensive collision during low-temperature adsorption. Further research is required to determine the exact cause in future works. Furthermore, the stability and sustainability of the optimized Pt/g-C_3_N_4_ are evaluated. A prolonged photocatalytic test over 22 h displays a slight reduction in hydrogen evolution following the initial 9 h, after which the rate stabilizes in the next 13 h (Fig. 4d). After 5 consecutive cycles, the hydrogen evolution capability only decreases by 9.6% (Fig. 4e). To analyze the mechanism for the slight decrease in catalytic performance, the Pt/g-C_3_N_4_ is characterized again after five cycles. The Pt content obtained by ICP-OES is 1.15%, which remains essentially consistent with the initial Pt/g-C_3_N_4_, indicating that Pt single atoms exhibit excellent anti-leaching properties. The HAADF-STEM image (Fig. S6†) shows that the Pt remains atomically dispersed after five reaction cycles, and no obvious change is observed in the FTIR spectrum (Fig. 5a). By comparing the XPS spectra before and after the catalytic reaction (Fig. 5b–e), the fine spectra of C 1s (Fig. 5b) and O 1s (Fig. 5c) remain almost completely unchanged, while the peaks of N 1s (Fig. 5d) and Pt 4f (Fig. 5e) both shift to lower binding energies. This suggests that the Pt single atoms have gained electrons and been partially reduced during the catalytic process, leading to a decrease in valence state. Slow-scan XRD (0.5° min^−1^) is employed to detect whether the reduction of Pt single atoms leads to the formation of metallic Pt. As shown in Fig. 5f, the low scan rate reduces the noise in the XRD pattern, clearly indicating that no peak corresponding to metallic Pt is present. Therefore, after prolonged catalytic reactions, the Pt atoms with lower valence state remain in an isolated state. The reduction in Pt valence state is attributed to the weakening of the metal–support interaction between the Pt single atoms and the g-C_3_N_4_ support,^35^ which can cause a gradual decline in hydrogen production performance. These results indicate that although anchoring Pt single atoms on g-C_3_N_4_*via* electrostatic adsorption provides good stability, the interaction between Pt and the g-C_3_N_4_ support tends to weaken over prolonged photocatalytic reaction time, a factor that should be addressed in future studies.

The improved laser synthesis method exhibits several distinct advantages over conventional laser synthesis approaches. Firstly, this method eliminates the need for high-energy lasers, which are typically required in traditional laser-based synthesis methods, thereby reducing equipment costs and power consumption. Secondly, this method uses water as the solvent. Compared to laser liquid-phase synthesis methods that employ organic solvents, it reduces solvent costs and the risk of environmental pollution. Thirdly, this method operates under low-temperature conditions, without the necessity for additional reductants or high-temperature treatments, aligning with green chemistry principles and minimizing energy consumption. Furthermore, this method is feasible for synthesizing highly dispersed single-atom catalysts, enhancing the utilization efficiency of noble metals. These advantages make the proposed method economically viable for large-scale production.

## 4. Conclusions

In summary, a novel synthesis strategy that involves synthesizing free-standing Pt single atoms and subsequently anchoring them on g-C_3_N_4_ has been successfully demonstrated. This innovative approach significantly boosts the photocatalytic hydrogen evolution performance. The integration of the freeze-phase photochemical method with the laser reduction technique facilitates the direct reduction of Pt precursors without the need for creating defects on the support material, overcoming the limitations associated with traditional high-energy laser synthesis methods. The resulting Pt/g-C_3_N_4_ SAC exhibits excellent photocatalytic activity and stability, highlighting the effectiveness of this synthesis approach in stabilizing single atoms and enhancing catalytic performance. This laser synthesis method also demonstrates the potential for anchoring single atoms on a variety of substrates, which will be further investigated in the future. This work provides a simple and eco-friendly method for producing high-performance SACs.

## Data availability

The data that support the findings of this study are available from the corresponding author upon reasonable request.

## Conflicts of interest

The authors have no conflicts to disclose.

## Supplementary Material

NA-OLF-D5NA00043B-s001
