# Supplementary material for: Pulsed laser synthesis of free-standing Pt single atoms in an ice block for enhancing photocatalytic hydrogen evolution of g-C3N4
Source: Nanoscale Adv. 2025 Mar 26;7(9):2474–82. doi: 10.1039/d5na00043b (PMC11959413; doi:10.1039/d5na00043b)
Supplement: NA-007-D5NA00043B-s001 [file NA-007-D5NA00043B-s001.pdf]

Supplementary Information (SI) for Nanoscale Advances.
This journal is © The Royal Society of Chemistry 2025

Pulsed laser synthesis of free-standing Pt single atoms in iced block for enhancing photocatalytic hydrogen evolution of g-C_3N_4

Yongming Fu,[*ab] Qianyu Lu,[ab] Jianhong Wang,[ab] Na Sun,[ab] Jinjun Gao,[ab] Peng Chen,[ab] Jizhou Wu[abc] and Jie Ma[*abc]

[a] School of Physics and Electronic Engineering & Institute of Laser Spectroscopy, State Key Laboratory of Quantum Optics and Quantum Optics Devices, Shanxi University, Taiyuan 030006, China

[b] Xinzhou Institute of Innovation Ecosystem, Shanxi University, Xinzhou 034000, China

[c] Collaborative Innovation Center of Extreme Optics, Shanxi University, Taiyuan 030006, China

[*] Corresponding authors. fuyongming@sxu.edu.cn; mj@sxu.edu.cn

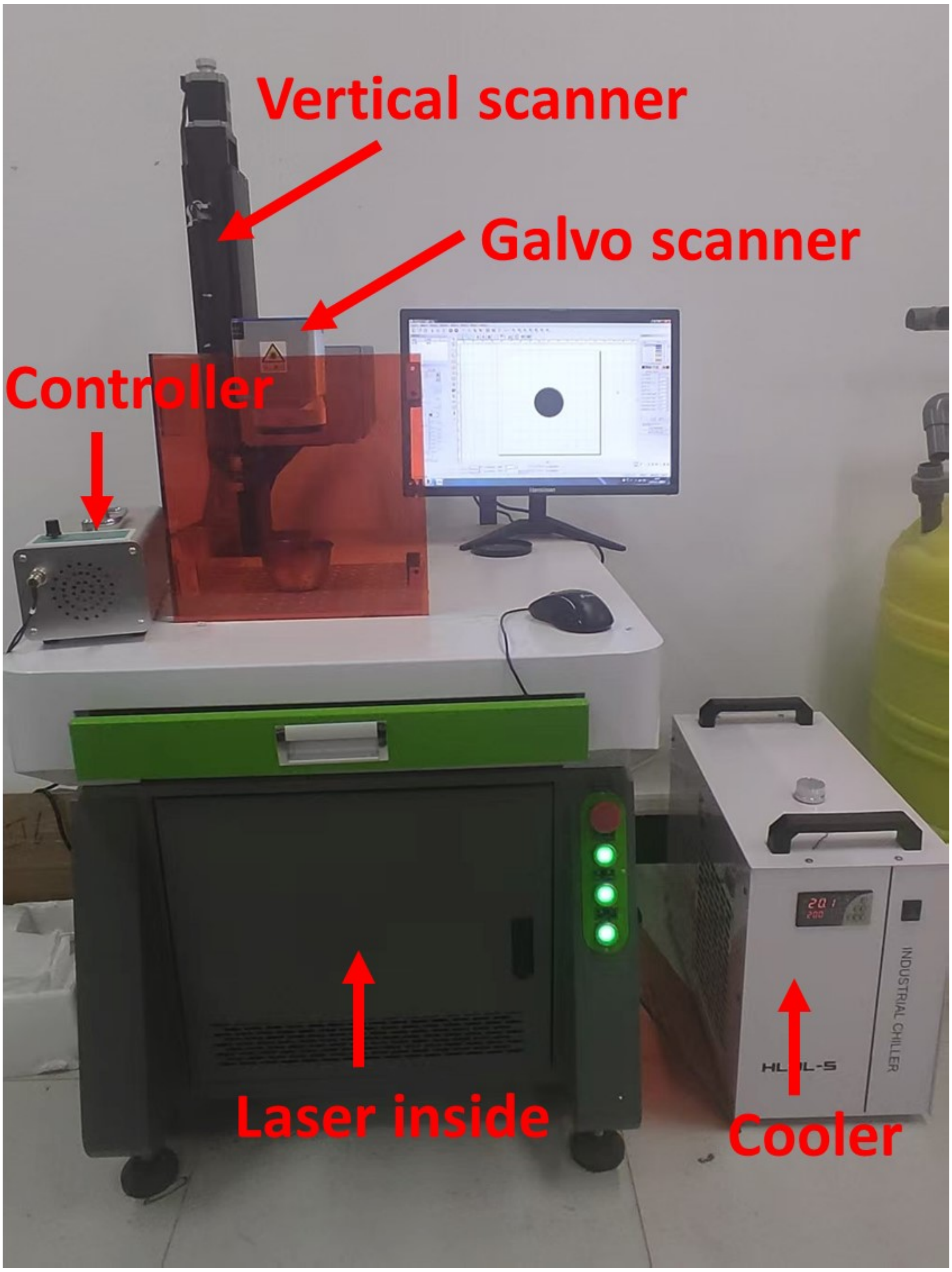

Fig. S1 The photograph of the laser scanning set.

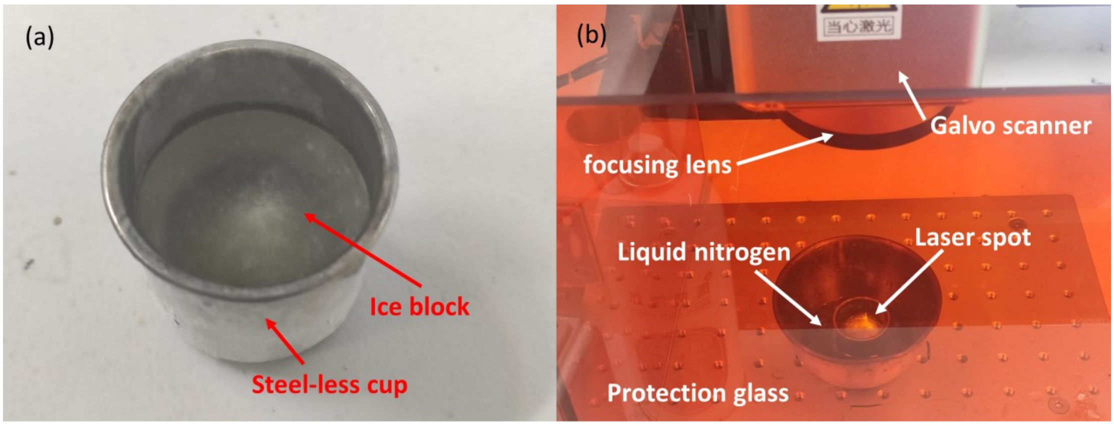

Fig. S2 The photographs of ice block in stainless-steel cup (a) and laser scanning process (b).

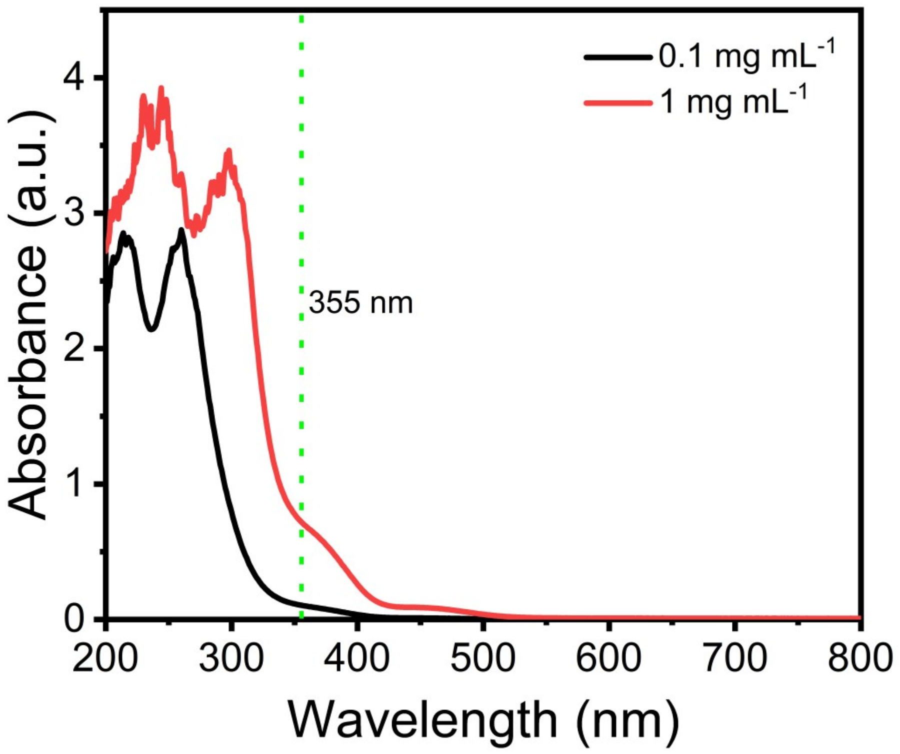

Fig. S3 The absorbance of the ice blocks with different H_2PtCl_6 concentrations.

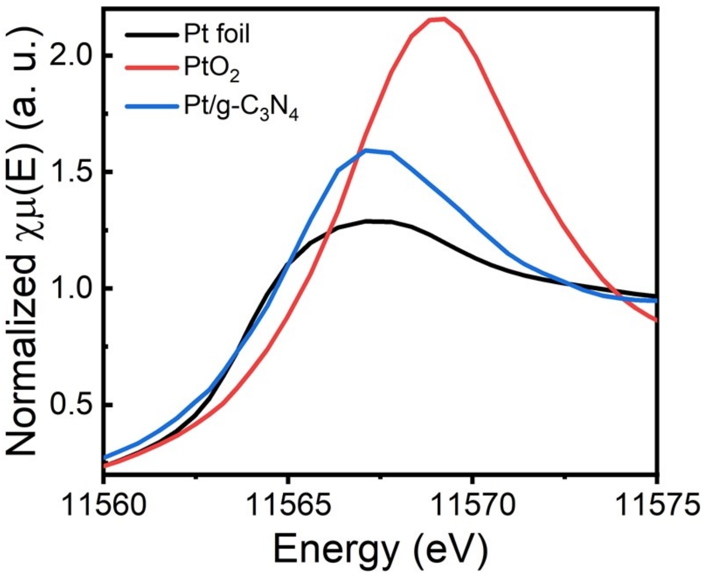

Fig. S4 The enlarged view of EXNES spectra.

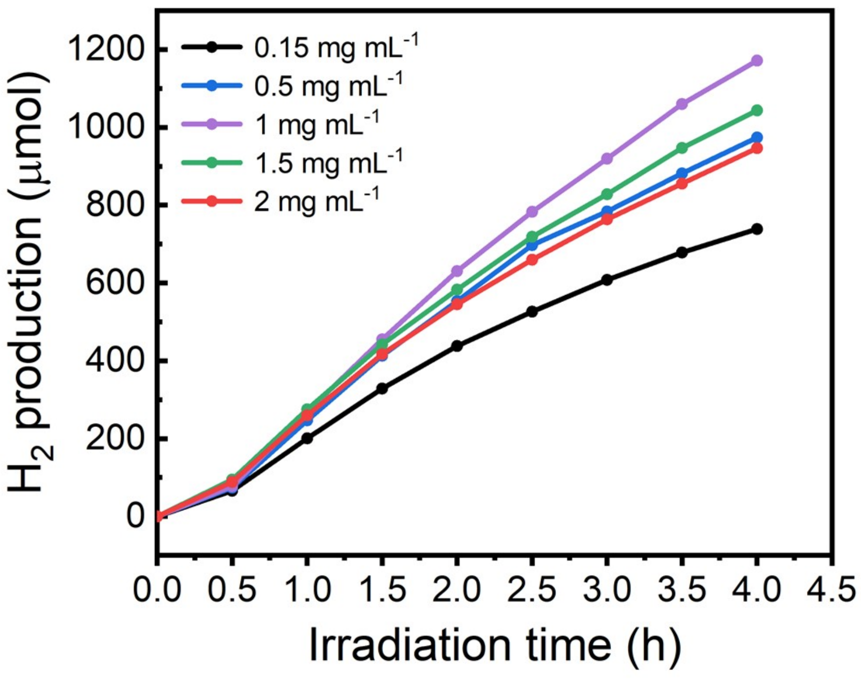

Fig. S5 The photocatalytic hydrogen production of Pt/g-C_3N_4 with different H_2PtCl_6 concentration.

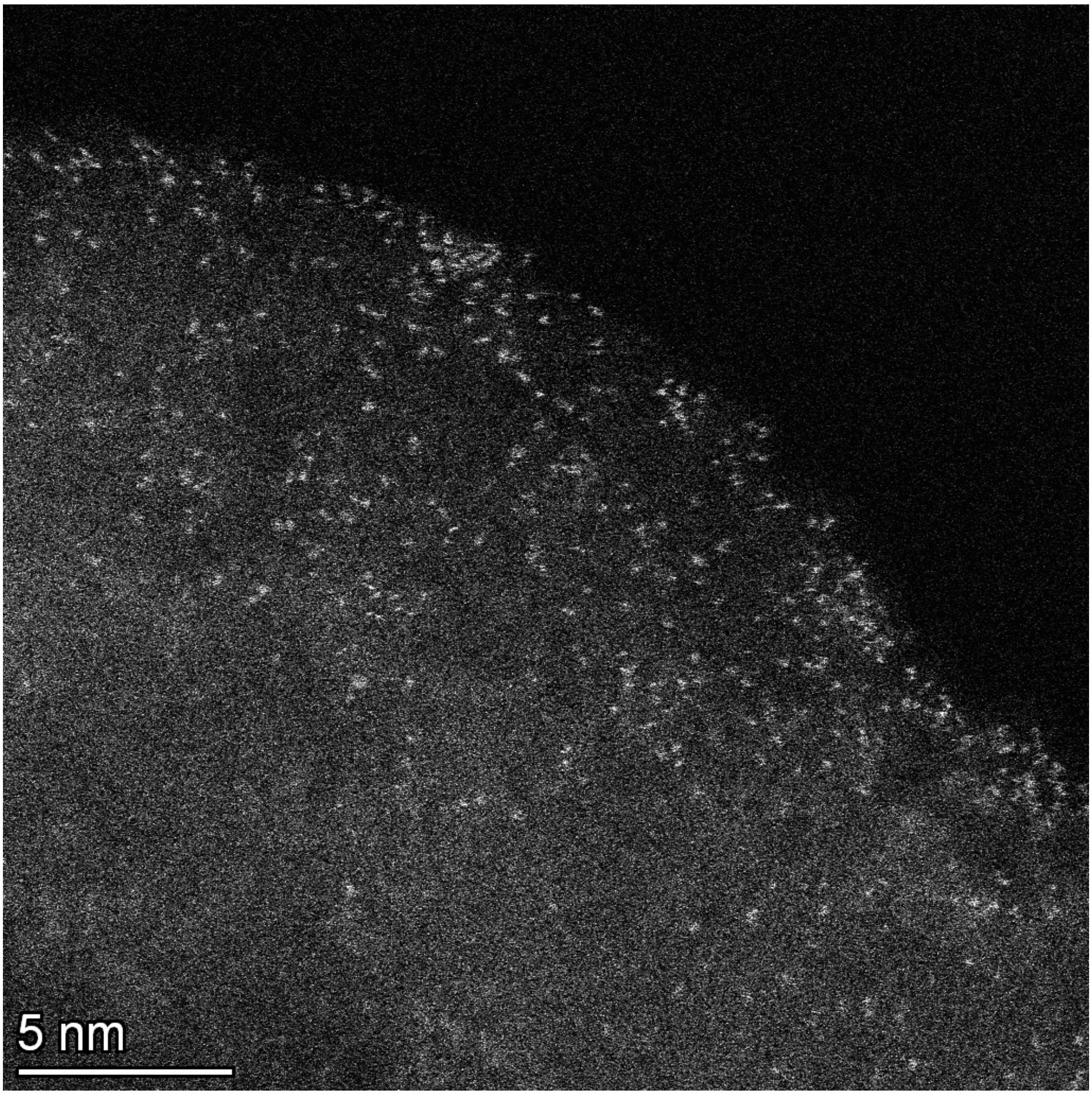

Fig. S6 The HAADF-STEM image of Pt/g-C_3N_4 after use.

Table S1. The precise fitting parameters of the EXAFS result.

**Sample**	**Bond type**	**CN**	**R (Å)**	**σ2 (10^{-3}Å^2)**	**R factor**
Pt/g-C_3N_4	Pt-N	3.8±0.2	2.25±0.01	2.3±1.6	0.012
Pt foil	Pt-Pt	12	2.75±0.01	6.7±2.2	0.006
PtO_2	Pt-O	5.9±1.2	2.03±0.01	1.9±1.6	0.011

CN: coordination number. S_0^2 was fixed to be 0.88 from Pt foil.

σ2: Debye−Waller factors
